# Integration of Utilities Infrastructures in a Future Internet Enabled Smart City Framework

**DOI:** 10.3390/s131114438

**Published:** 2013-10-25

**Authors:** Luis Sánchez, Ignacio Elicegui, Javier Cuesta, Luis Muñoz, Jorge Lanza

**Affiliations:** Communications Engineering Department, University of Cantabria, Edificio de Ingeniería de Telecomunicaciones, Plaza de la Ciencia s/n, Santander 39005, Spain; E-Mails: iemaestro@tlmat.unican.es (I.E.); jcuesta@tlmat.unican.es (J.C.); luis@tlmat.unican.es (L.M.); jlanza@tlmat.unican.es (J.L.)

**Keywords:** future internet, smart city, internet of things, proof-of-concept, key performance indicators

## Abstract

Improving efficiency of city services and facilitating a more sustainable development of cities are the main drivers of the smart city concept. Information and Communication Technologies (ICT) play a crucial role in making cities smarter, more accessible and more open. In this paper we present a novel architecture exploiting major concepts from the Future Internet (FI) paradigm addressing the challenges that need to be overcome when creating smarter cities. This architecture takes advantage of both the critical communications infrastructures already in place and owned by the utilities as well as of the infrastructure belonging to the city municipalities to accelerate efficient provision of existing and new city services. The paper highlights how FI technologies create the necessary glue and logic that allows the integration of current vertical and isolated city services into a holistic solution, which enables a huge forward leap for the efficiency and sustainability of our cities. Moreover, the paper describes a real-world prototype, that instantiates the aforementioned architecture, deployed in one of the parks of the city of Santander providing an autonomous public street lighting adaptation service. This prototype is a showcase on how added-value services can be seamlessly created on top of the proposed architecture.

## 1. Introduction

Urban infrastructures had to be extended and changed from their original construction due to the constant development and growth of our cities. Notably, the core of these installations often still dates back to their origin (e.g., the London sewage network is still partially built on a roman legacy) with some on demand extensions which proved sufficient for providing citizens with the necessary services, at least so far.

The growth and change in cities is accelerating and makes it even harder to provide a sustainable urban living environment [1]. The use of an Information and Communication Technologies (ICT) based infrastructure alongside the traditional utilities and services infrastructures will be the next big step in the development of cities [2,3]. Information systems will help to optimise infrastructure, inform citizens and build a communication network that spans the globe allowing tailoring the utility and services delivery to the actual needs rather than to overprovision for peak demands; in turn, the confluence of ICT and city services will fuel economic growth and prosperity and will form new city ecosystems. This revolution is still only at the beginning as suitable infrastructures are being deployed and significant investments into the city infrastructures are made.

However, the scale of all systems mentioned above is expanding: the number of vehicles in the public transportation system, the number of waste bins that need to be collected and amount of waste removed, the length of the water distribution pipes, the extension and complexity of public lighting infrastructure, *etc.* To ensure sustainable development, it is crucial to safeguard efficient flow of information between the domains and stakeholders and to enable seamless sharing of the deployed infrastructure regardless of the entity or individual who deployed it.

Before presenting the functional architecture and describing the building blocks that compose it, we provide a thorough review of which have been the main considerations motivating the final design. In this sense we have identified the following five design principles: (i) integration of heterogeneous infrastructure; (ii) IoT-like large-scale network; (iii) dynamic network management; (iv) Sensing as a Service; and (v) cloud of services.

Therefore, in this paper we are proposing the smart city community to leverage an innovative Future Internet (FI) enabled architecture aiming at making the traditional utilities'; services and infrastructure accessible by the non-usual stakeholders (e.g., telco provider, enterprise or 3rd party developers). This approach will enable the creation of more transversal and smarter services. Such architecture is additionally biased by the following conditions:

(1) It relies on the FI paradigm for providing ubiquitous services to the users in the cities;
(2) It exploits the infrastructures belonging to the utility providers for improving the quality of urban services while reducing the cost of deployment of new infrastructure and avoiding duplicity.

In order to demonstrate the feasibility of the solution proposed and to assess the potential gains enabled by such smart city platform we have developed a real-world prototype that is also described in this paper. The proof-of-concept prototype description includes not only the use case presentation but also the specification of the deployment done in one of the parks in the city of Santander. Several wireless sensor devices have been integrated in this deployment. Results from the assessment of some Key Performance Indicators (KPI) evaluated for the prototyped use case are provided.

This paper is organized as follows: Section 2 describes related work on smart city research, on the current practices on utility services provision, and on FI platform development. The design principles that have been taken into account are sketched in Section 3. Section 4 describes the smart city platform architecture that we are proposing. In Section 5 we present the proof-of-concept prototype that has been implemented following the designed framework guidelines. Both the physical deployment carried out as well as the autonomous light intensity adaptation service that has been developed will be thoroughly described. Finally, Section 6 concludes this paper.

## 2. Related Work

In this section we present some of the most representative works regarding: (i) smart city platforms; (ii) utilities infrastructure integration; and (iii) Future Internet enablers for smart cities.

### 2.1. Smart City Platforms

Smart city paradigm is gathering a lot of attention lately. Still, definitions vary largely and there is a lack of models to guide their design. Giffinger *et al.* [4] has identified six dimensions of a smart city: smart economy; smart mobility; smart environment; smart people; smart living; and, finally, smart governance. Thus, it is possible to find multiple definitions and initiatives targeting the smart cities. Although many of them do not necessarily consider ICT as implicit pre-requisites for the system design we are herewith considering those initiatives which are based on technological developments.

Cisco Smart + Connected Communities [5] uses intelligent networking capabilities to weave together people, services, community assets, and information into a single pervasive solution. “Smart + Connected” acknowledges the essential role of the network as the platform to help transform physical communities to connected communities. It also encapsulates a new way of thinking about how communities are designed, built, managed, and renewed to achieve social, economic, and environmental sustainability.

IBM's Intelligent Operations Center for Smarter Cities [6] provides an executive dashboard to help city leaders gain insight into all aspects of the city. The executive dashboard spans agencies and enables drill-down capability into each underlying agency such as emergency management, public safety, social services, transportation, or water.

Telvent's Integrated City Management Platform (ICM) [7] targets an integrated system-of-systems. The ICM platform integrates the various systems within the city, exchanging information through a common platform between the agencies which need it. The ICM platform also includes a suite of analytics, business intelligence, and decision support capabilities which interpret the data collected from infrastructure systems into actionable intelligence. Currently ICM platform provides the Smart cities vision in a custom premise deployment at cities, and is mainly oriented towards municipality decision support for the transport and water domains but in an isolated instantiation of the platform.

LIVE Singapore [8] is developing an open platform for the collection, the combination and fusion as well as the distribution of real-time data that originate from a large number of different sources. It provides people with access to a range of useful real-time information about their city by developing an open platform for the collection, elaboration and distribution of real-time data that reflect urban activity. Giving people visual and tangible access to real-time information about their city enables them to take their decisions more in sync with their environment, with what is actually happening around them.

Contrarily to the channels of access that we can find on these platforms, the smart city platform we are proposing builds an ecosystem through which smart city innovation will be catalysed. Not only city authorities or utilities will be given access to the platform but citizens (SMEs, entrepreneurs and individuals) will be empowered to build innovative services for each other and the city on top of these smart city platforms without the need to stroll over a multitude of different interfaces. A repository of services, including dashboards and reports to provide immediate access to the details of the operational services, will be provided thus enabling re-usability and co-creation.

### 2.2. Utilities Infrastructure Integration

In a smart city, energy, water, transportation, waste management, and other key services are managed by different utilities that manage their own infrastructure. Significant levels of automation, communications and information technology are already being brought. There is a clear movement toward driving more intelligence into field equipment to make faster decisions on fault isolation, location and restoration, reconfiguration, and management of the complex system these utilities control.

The smart grid concept [9,10] has been the most analysed of the utilities'; infrastructures but the work in this field has been mainly focused on improving the efficiency on electricity delivery and grid management. Geellings [11] discussed on a platform that enabled the evolution of power system into a highly interconnected, complex, and interactive network of power systems, ICT and electronic commerce applications. Ipakchi and Albuyeh [12] analysed several challenges for the grid of the future with emphasis on demand response as well as distributed generation and storage capabilities. However, there is no reference to the need of integrating power systems into a higher level platform aimed at orchestrated optimization. To the best of our knowledge, the appropriate tools have not been already developed to fully exploit the infrastructure of city utilities.

### 2.3. Future Internet Enablers for Smart Cities

FI-WARE [13] is currently delivering a holistic infrastructure, built upon elements (called Generic Enablers) which offer reusable and commonly shared functions to facilitate the development of Future Internet applications in various sectors. The enablers are organised according to technical layers (also known as chapters) such as IoT layer, data/context management layer, security plane, service/application ecosystem, cloud layer and networks layer. Enablers from these chapters are being exposed via an enabler catalogue that will let others to test and validate those enablers for their specific needs.

Additionally, the European Commission is currently funding thirteen smart cities projects under the 2010 and 2011 Competitiveness and Innovation Programme (CIP) Objective: “Open innovation for future Internet-enabled services in smart cities” [14]. The main and common goal of these projects is to help cities deploy ICT in new innovative ways that enable them to become ‘smarter’. However, these projects adopt the traditional concept of building tailored systems conceived on customised equipment and software.

In contrast to these projects, the approach followed in the proposed platform is to adopt generic future internet enablers that can be reused, complemented and tailored by other application domain specific enablers. The advantage of this approach is that core functionalities like network management, data access, data processing, service repository, service provision environment, *etc.* are common and can be reused in different application domains, as well as in the different platform instantiations within one domain.

## 3. System Design Considerations

Cities are complex systems, a conglomerate of people, organizations, businesses, city infrastructures and services, and more recently deployments of smart devices like sensors and actuators. As all complex systems, cities need to be managed in order to ensure uninterrupted performance of all relevant activities and thus uninterrupted living conditions for all stakeholders.

The coordination of all these activities and domains is of paramount importance to ensure efficient and effective city services management. However, in modern cities the coordination is usually not done on a daily basis, but more on a strategic level and mainly from the administrative and political perspective with poor real-time feedback. Each public service is often run as a standalone activity preventing efficient exchange of information and sharing of available infrastructure.

**Figure 1.**
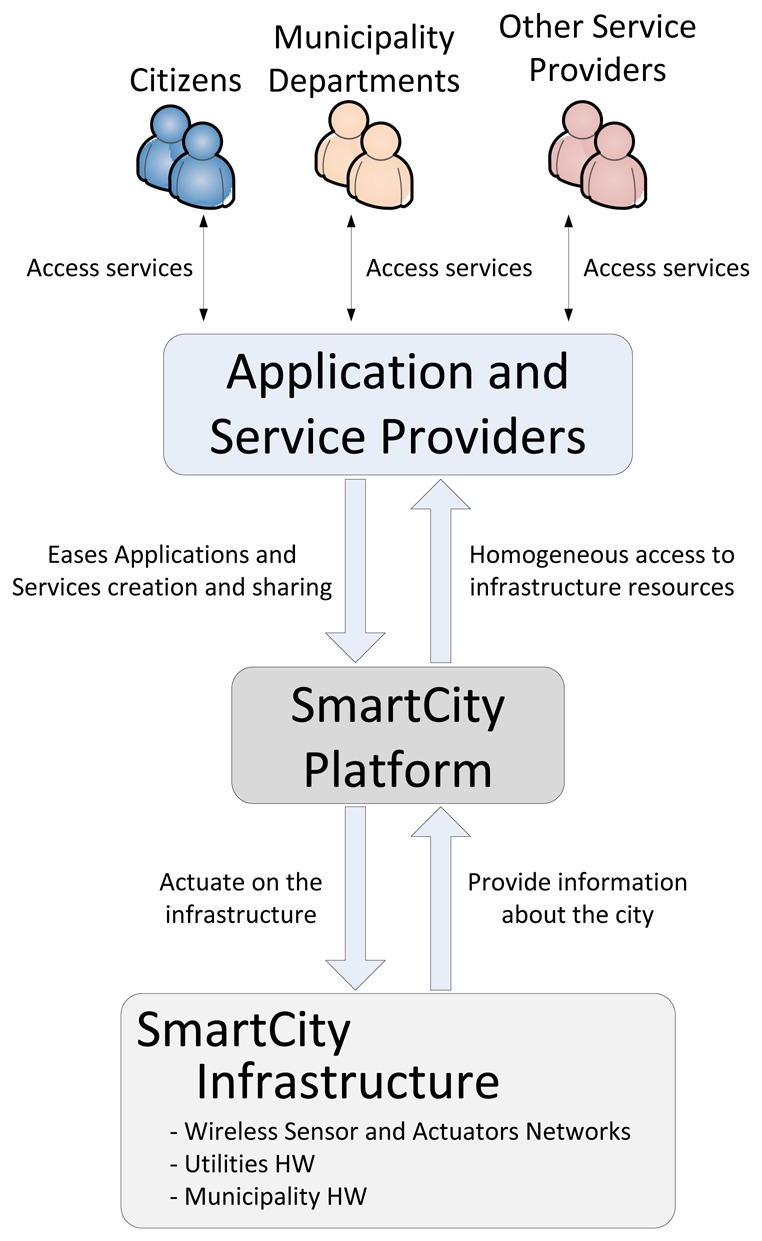
SmartCity actors interactions.

To support the creation of more transversal and smarter services the development of ICT architecture meeting the smart city ecosystem requirements becomes crucial. Figure 1 shows the high-level interactions between the main actors involved in the smart city scenario. The key aspects that have to be highlighted are the fact that existing and future city infrastructure has to be managed in a homogeneous way in order to fully exploit its potential. Additionally, it is mandatory that the creation of services extracting value from the huge amount of information becomes much easier. Finally, it must be possible that already available services could be re-used for creating new ones. In this sense, multiple interactions (e.g., C2B, B2B) should be supported in a transparent manner.

Taking all this into account we have considered the following aspects as the main items gathering the majority of system requirements to be addressed by the smart city framework that we are proposing.

### 3.1. Integration of Heterogeneous Infrastructure

The diversity of devices and networks that are available is the first aspect that needs to be addressed. This heterogeneity of device classes and device technologies should be homogenized in order to be integrated with other FI infrastructure and exported through the proposed architecture. The appropriate interworking functions have to be instantiated at both the communication plane and the management and data planes.

It is important to note that not only the application domains to which this infrastructure belongs will be diverse but also the nature and technological footprint of the devices within the same application domain might be also heterogeneous. In this sense, at the heart of the public services systems, data can be collected by old Programmable Logic Controllers (PLC) via a Supervisory Control and Data Acquisition (SCADA) computer system as well as by the latest hardware and software for analysis, monitoring and billing including the latest mobile devices for work in the field.

Last but not least, new ways for getting information on city status are starting to be employed from large-scale sensor networks deployments [15] to various sorts of crowdsourcing [16,17] or the multitude of surveillance cameras [18]. The importance of this new sensing and actuating infrastructure examples is twofold. On the one, hand it increases the heterogeneity. On the other hand, it is a clear indication that the trend is towards yet more diverse environments that must be supported on the most seamless manner by whatever solution is proposed.

### 3.2. IoT-Like Large-Scale Network

Several definitions of IoT can already be found within the research literature [19–21] but they have been evolving towards the basic idea of the pervasive presence around us of a variety of things or objects which are able to interact with each other and cooperate with their neighbours to reach common goals [22].

Smart cities are clear examples of this paradigm, where a plethora of devices interact and can serve to different common goals. In this sense, the main challenges for IoT platforms are present in the scenario we are tackling from the fact that a network with such a large amount of devices has to allow transparent access both from and to all the nodes. It is particularly important to achieve a sufficient level of abstraction that hides the underlying complexity of the physical network. Additionally it is also critical to support self-organization of the underlying infrastructure so that services provided on top of it are not interrupted upon network failures.

### 3.3. Dynamic Network Management

In order to meet the challenging task of developing a dependable smart city platform it is necessary to implement robust techniques for realizing out-of-band management and control planes. Under normal operation, such a platform is in a constant state of flux. New sensor devices are detected and registered with the platform. The context status parameters (battery level, CPU utilisation, memory consumption) of existing devices change.

Additionally, there are dynamic variations to network context and to application requirements over the time. Node membership of the network changes as new nodes are added or fail (due to power outage or hardware failure) or, are disconnected (due to transient connectivity in the case of mobile nodes). Individually, each IoT node may transition through a number of possible states during the operation; the responsiveness of a node to issued commands depends on its current state. Further, supporting multiple application domains introduces dynamic variations in the spatial and temporal characteristics of sensor data based on the new requirements of developed applications and services. These applications targeting different domains may even share the IoT devices generating their required sensor data streams concurrently.

Finally, the sheer number deployed of IoT devices and dynamicity inherent in these deployments, adds special significance to the management of this kind of infrastructures. With the scale and variety of testbed management events to track, one cannot assume human intervention alone is sufficient to provide timely response to events and remediation to faults; a certain degree of automation is required, keeping the human in the loop only for decision-making and policy-specification.

### 3.4. Sensing as A Service

The classical approach in which city services are provided in a vertical manner has to be abandoned to avoid further creation of isolated silos where innovation is limited. Sharing information and infrastructure across services will enable cooperation and will allow the exploitation of synergies, at least in terms of data collection and exploitation. Teaming up city service infrastructures will be able to correlate different information sources and will allow a new much more efficient and sustainable ecosystem.

Sheng *et al.* [23] proposed the Sensing as a Service (SaaS) concept as a way for building applications based sensing services using mobile phones via a cloud computing system. In order to fully exploit the smart city concept as it has been described above, it is necessary to expose all the sensing and actuating possibilities in the city. This must be done on a transparent and open way that eases the development and provision of applications. The smart city framework must provide these interfaces. They must fulfil application developers' demands independently of the device providing the actual information being consumed and without being affected by infrastructure dynamics.

### 3.5. Cloud of Services

Beyond the provision of more efficient and effective urban services the smart city framework has to provide additional value. A city is not only for living, but also for economic activities. Hence, the smart city infrastructure has to be part of the economic fabric of a city and has to stimulate creation of new technologies and services by individuals or businesses. Thus, it is necessary to provide tools to developers that can help them to build new applications and services. Additionally, it should be possible to generate new applications and services from software artefacts already available, creating new services by composition with those already existing.

To tackle these issues, it should be possible to leverage cloud computing concepts as enablers for building a real service ecosystem where the following functionalities are provided: (i) services could run and access data capabilities offered by the underlying infrastructure; (ii) tools that allow creation of services able to exploit available capabilities; (iii) tools that make all the actors involved aware about the services being available in the ecosystem; (iv) components and tools that make possible to measure and control the consumption of the service provided; and (v) tools that simplify the end user access to the information available. The combination of the Cloud and SaaS paradigms enables multiple ways of interaction among the smart city stakeholders. This way Business-To-Consumer (B2C), Business-To-Business (B2B), Consumer-To-Business (C2B) and Consumer-To-Consumer (C2C) relations are supported.

## 4. Architecture Overview

**Figure 2.**
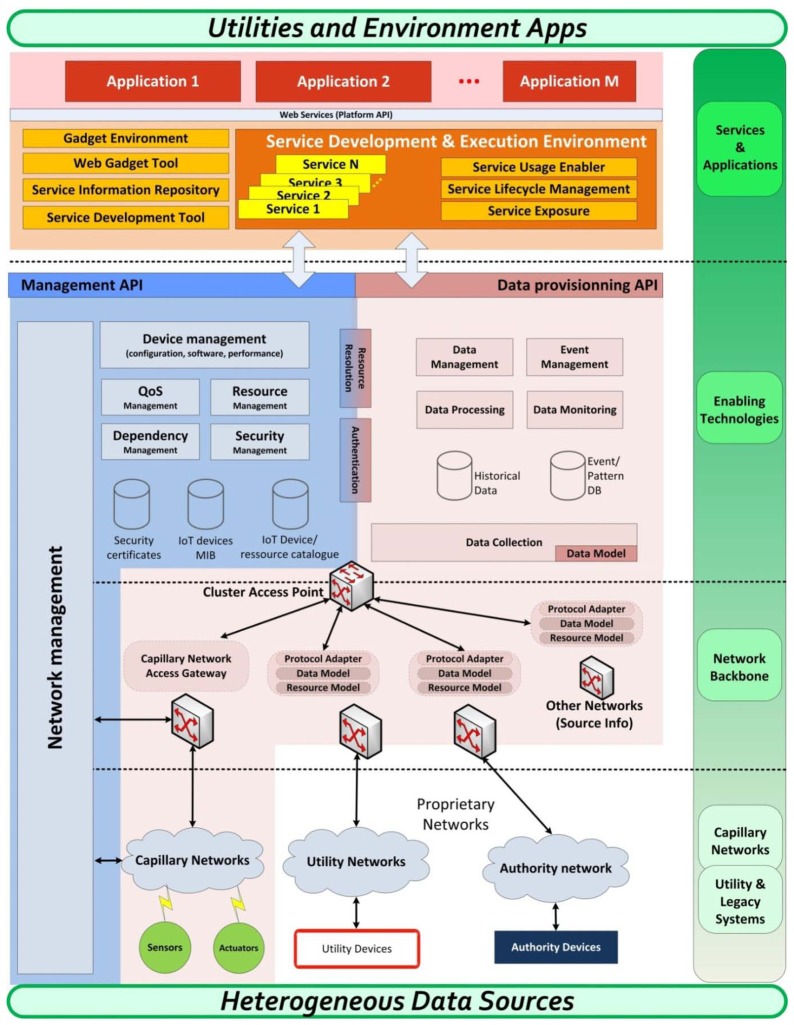
Smart City platform high-level functional architecture.

Taking the above mentioned design considerations the architecture shown in Figure 2 is proposed.

This architecture is realised by a four tiered approach, which consists of:

- Capillary networks tier, comprising the heterogeneous underlying infrastructure composed of wireless sensors and actuators as well as already existing devices with sensing and actuating capabilities that belong to utilities and local authorities.
- Network backbone tier, which interconnects every capillary network and homogenizes the access to aforementioned heterogeneous infrastructures.
- Enabling technologies tier, which holds future internet components exporting infrastructure capabilities on a uniform manner.
- Services and applications tier, where platform services are offered to application developers.

Through this group of layers, one of the main targets of this proposed architecture is to provide access to application developers to the set of heterogeneous data supplied by different sensor networks and legacy systems, in an open and standardized way, completely abstracting these two worlds. The different functionalities offered at each of these tiers are presented in the following sub-chapters.

### 4.1. Capillary Networks and Utility Legacy Systems

At the bottom of the architecture, a heterogeneous infrastructure is assumed. It is important to highlight that this layer provides the necessary substrate consisting of IoT devices and the communications networks that allow bidirectional access to the underlying infrastructure.

The key aspect that differentiates this architecture from other existing IoT reference models [24] is that it explicitly incorporates the utilities and local authorities' networks as part of it.

### 4.2. Enabling Technologies

If the network backbone layer provides the ways to collect data and control the nodes from the capillary networks and legacy systems, the set of enablers on this tier will focus on the data mining and infrastructure management, providing functionalities related with the data and the management plane. It makes up the core of the proposed architecture, providing also the tools for the upper layer to access data and nodes capabilities.

Related to management plane, the main functionalities provided at this tier are:

- To support registration of IoT devices (sensors/actuators) and IoT gateways.
- To enable the backend to control and access information from IoT gateways and IoT devices.
- To provide access to information of the different devices registered into the platform (status, location, manufacturer, measurements…) upon request from external management applications.

In the data plane, focused on data collection and storing, the basic functionalities provided are:

- To support measurements collection and storage processes for different phenomena.
- To provide mechanisms for upper layers applications to access data collected (historic data and last measurements).
- To enable the submission of commands to devices.
- To implement a publish-subscribe-notify model, allowing rules for subscription to specific events, conditions or devices and the definition of alerts.

The definition of this layer and the enablers initially included have been done following the FI-WARE project guidelines [13] which, aside from giving a more standardized and portable vision of the whole architecture for smart cities, allows that new enablers can be easily integrated into a final deployment, adding new functionalities as event processing, big data analysis and so on.

### 4.3. Service and Applications Layer

Based on the interfaces given by the enabling technologies, this tier provides with the development tools needed to create new services which exploit the information gathered from the sensor networks and utility systems, as well as allowing interaction with the deployed actuators.

It also includes the runtime environment with the application server responsible for executing these services and the set of plugins and enablers (service repository, operative systems oriented tools, development libraries, *etc.*) to complete the service infrastructure.

Additionally, service store like enablers are supported so that it is possible to create and exchange services on a mash-up fashion.

This new set of services is created and supported by the smart city, which is responsible of security when accessing to its data or manage its networks, and defines the user interface provided to application developers. This interface is completely independent from the protocols on the data gathering layers so allows creating new open and portable final applications to improve citizen's life quality in smart cities.

Finally, it is important to highlight that services provide another abstraction level to application developers which can reuse the functionalities provided by these services in an abstracted manner while generating applications with added-value for the end-users. The platform treats these services as abstract entities thus enabling interactions between the actors on a complete transparent way.

The way services are exported and the creation of a services environment that enable transparent composition and re-composition of services enables different stakeholders to interact. This way, traditional interactions between service providers and service consumers is greatly enriched as the service ecosystem is opened to a wider range of actors.

## 5. Autonomous Public Street Lighting Adaptability System Prototype

### 5.1. Use Case Description

The Santander City Council spends every year around 3.5 M€ in street lighting. This expenditure, together with urban transport, water and gas distribution, waste management and infrastructure maintenance is one of the main, and key, public services for the city.

Nowadays, illumination levels at city streets are regulated by electric power controllers. Every of these controllers supplies electrical power to a defined set of lampposts. They operate based on an astronomical clock which gives, depending on date and location, the sunset and sunrise times. In its most standard working mode, the device automatically turns on (maximum power supplied → maximum light level expected) its streetlights when sunset comes and turns off them at sunrise.

Santander City Council is promoting an upgrade of its controllers by adding an intelligent lighting control panel, able to collect data from the power controller and operate with it, connected to a communication module (GSM/GPRS/UMTS capable) which sets up remote reading and controlling functions.

The *Autonomous Public Street Lighting Adaptability* use case aims at increasing the public streetlight service efficiency by automatically adapting the power supplied to the lampposts and their corresponding light level to the current needs of the area covered.

**Figure 3.**
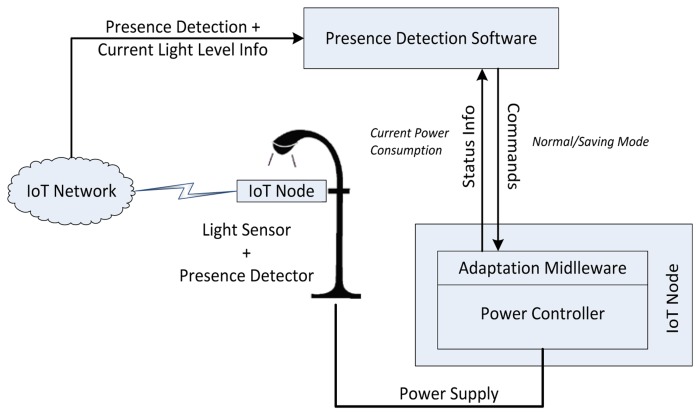
Use case interactions diagram.

Depending on the city area and the main use of the streetlights, the phenomenon (or set of parameters) chosen to increase/decrease the illumination level may vary (e.g., weather conditions, existing illumination levels, vehicular traffic, *etc.*). As it is shown in Figure 3, the prototype deployed is focused on pedestrian presence; this is, once the streetlight has been turned on (to its normal level), if no pedestrian presence is detected, the power supplied by the controller will be set to “saving mode” and the light level will decrease. When presence is noticed, automatically the command to activate the normal mode again is sent to the controller, till no one is detected in the area. This operating mode requires a sensor network including presence detector nodes to be deployed in the area covered by the prototype. These nodes will also build in a light sensor, intended to measure the ambient light level. The information provided by this sensor will be used in a twofold manner: when ambient light falls below a fixed level (e.g., due to weather conditions), regardless of the astronomical clock of the power controller, the streetlights can be switched on and presence detection mechanism be activated; apart of this, the light sensors installed covers a range from 0 to 100 lux, which makes them suitable (properly positioned) to evaluate light pollution.

### 5.2. Prototype Architecture

**Figure 4.**
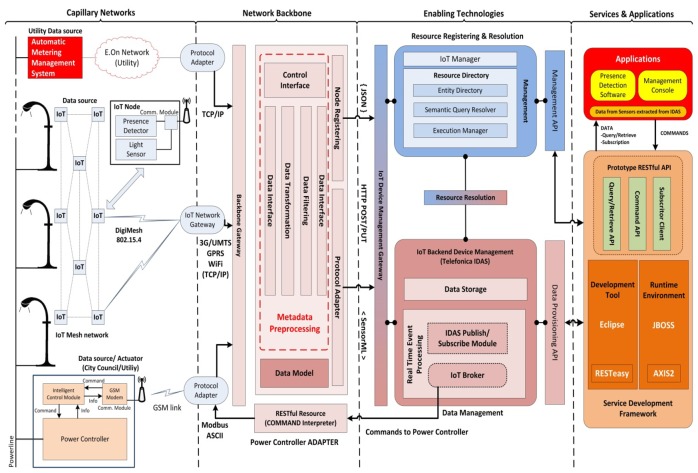
Deployed prototype architecture.

The prototype architecture consists on a concrete implementation of the general proposed one in Section 4. Figure 4 shows the realization of the prototype based on the generic architecture. The different components implemented at each of the reference model tiers are presented in the following sub-chapters.

#### 5.2.1. Capillary Networks Prototype Components

In this tier the utility infrastructure (power controller and Advanced Metering and Management System—AMMS), the wireless sensor nodes and the power controller are included.

The AMMS device belongs to E-On Spain (electric power provider) and is one of the data sources used in the prototype. To get this data, a SSH connection is established through the utility network. Information gathered at the AMMS is adapted and directed to the Metadata Preprocessing. It uploads daily information about active and reactive power consumption.

IoT Nodes include two kind of sensors: one light intensity sensor and one presence detector. The first one provides illumination level in luxes. The second one triggers an event upon pedestrians are detected. Ten of these nodes have been deployed around the pathways in the park. They use a wireless communication module based on IEEE 802.15.4 to create a mesh network in which all cooperate for relaying the information towards the IoT Network Gateway. This latter one sends all data collected from nodes to the Metadata Preprocessing component, using a 3G connection, passing through the Backbone Gateway.

The Intelligent Control Module of the Power Controller is part of the local authority infrastructure. It gathers data on instantaneous energy supplied (active power—W and reactive power—VAr, total voltage—V and total electric current—mA) and reports it to the Metadata Preprocessing block. It also processes and executes all commands received (e.g., commands to switch on/off the streetlights or to change to normal/saving mode). Due to the scale of the prototype, only one power controller is involved (identified as *RG_LAS_LLAMAS_01*). It is equipped with an intelligent control unit and a communication module supporting Modbus ASCII [25] data protocol over GSM connection. The protocol adapter created to connect with the power controller uses a GSM modem to convert from Modbus ASCII to TCP/IP and reach the Backbone Gateway.

#### 5.2.2. Network Backbone Prototype Components

There are three components implementing network backbone functionalities.

The backbone gateway works as the point where all data sources must send their observations. The backbone gateway receives data from different sources and in different formats. The AMMS works with text comma-separated-value files; IoT mesh network sends proprietary-formatted frames; and the power controller receives, processes and sends through a Modbus ASCII channel. This data must be adapted to be correctly accepted by the corresponding enabler on the next layer. The IoT Backend Device Management module process observations in SensorML (XML) while and the Resource Directory uses JavaScript Object Notation (JSON) for nodes' registering and management.

Metadata Preprocessing module stores information from both, observations and node capabilities according to a specified data model. The core of this component is a MySQL database manager, where all data coming from the devices is checked and stored. Three software blocks implement its functionalities: (i) *Data Interface (in)* + *Data transformation*: reads the data files, receives the data frames or accesses the external Data Bases (DB), collects the information and stores it in the core DB, according the internal data model; (ii) *Data Filtering* + *Data Interface (out)*: composes the messages carrying the information, using the data stored in the core DB, creates the SensorML file or the JSON file, establishes the connection with the corresponding component in the upper tier and sends the messages, using HTTP POST/PUT, as required by these enabling technologies components; and (iii) *Control Interface*: based on the MySQL capabilities, implements the functionalities and interfaces to control this asset, in order to modify the data model, add new data interfaces or monitor the data sent.

Power Controller Adapter has been implemented as a middleware that abstracts the proprietary protocols and mechanisms of the advanced power controllers used in this deployment. This middleware implements a RESTful API that provides access to controller's information and commands to third parties' software, turning this legacy device into an IoT Node that can be dealt with as a sensor and/or actuator and be easily integrated into FI and smart cities deployments. Upon reception of the commands from the upper tier (as PUT and GET web services), the adapter implements the command conversions to be sent to the power controller in its Modbus protocol.

#### 5.2.3. Enabling Technologies Prototype Components

The IoT chapter of the core platform developed in FI-WARE project has been used as the baseline for realizing this tier in the prototype. This alignment ensures our prototype to be scalable, portable and compatible with other sensors, services and functionalities. Two enablers, from the FI-WARE catalogue have been used in our prototype.

The Resource Directory asset, instantiated on the prototype server, registers all information about nodes and their capabilities. This information is used to implement management and security functions, establishing who and how nodes can be reached and modified, also changing the network configuration. In addition, it implements resource discovery mechanism, which automatically allows the platform to add new devices or discard those nodes that are not working.

The IoT Backend Device Management generic enabler, implemented by an instance of the Telefonica IDAS [26], collects and stores all observations sent by the capillary networks through the IoT Backend Device Gateway. Using the Resource Resolution block, this element can access to the data stored in the Resource Directory instance and interact with it. The Backend Device Management enabler provides a RESTful API for the service development layer to access the current and historic data supplied by nodes in the capillary network (the IoT broker manage all these requests). It also allows registering a service to a node or a set of data (through the publish/subscribe element), so a notification can be sent by the enabler when any change is raised (e.g., when a pedestrian is detected—service registered to presence data). This API also allows sending commands if the final node has been registered as an actuator (e.g., authorized services can operate on IoT Nodes—activate the power controller or change its operating mode).

#### 5.2.4. Enabling Technologies Prototype Components

The functionalities provided by the IoT Backend Device enabler's API are exploited at this tier. A set of specific services are created to allow third parties to develop applications based on data and capabilities offered by the deployed capillary networks and legacy systems. In order to support scalability, composition and standardization, this layer has also been developed following the guidelines established in FI-WARE for applications and services delivery framework. In practice, the prototype includes two basic blocks in this tier:

*Eclipse 4.2* (Juno version) for Java EE developers, complemented with JSON and JBoss plugins realizes the service development framework.
*JBOSS Application Server* 7.1. with RESTEasy support realizes the runtime environment.

The set of services that was meant to be offered to application developers was packed as a RESTful API that provides the following services, returning the information in JSON or XML format:

- Light Resources API
  ○ *Node_Current_Status*: Retrieves last light level (+ battery status) sent by the node.
  ○ *Line_Current_Status*: Retrieves last light level measurements sent by the nodes belonging to the Line identified (nodes are assigned to a streetlight line, belonging to a power controller).
  ○ *Node_Historic*: Retrieves the historic measurements (light levels) of the Node identified, between the dates (and time) pointed or between the date (and time) pointed and current date & time.
- Presence Detection API
  ○ *PD_Current_Status*: Retrieves last measurement (Presence Detection + battery status) sent by the node.
  ○ *Subscriptor_Service*: Subscribes an URI to IDAS' Presence Detection Service (HTTP POST). The URI subscribed will receive a notification when a presence detection observation is produced.
- Regulator Resource API
  ○ *Reg_Current_Status*: Retrieves current measurements and status parameters from the Power regulator pointed (RG_LAS_LLAMAS_01).
  ○ *SET* (Command): Sends (HTTP GET) a command to the Power Regulator signed (RG_LAS_LLAMAS_01). The commands currently supported are “Data” to retrieve data form controller; “Power” to set lighting level to top/Switch ON the lights; “Saving” to set lighting mode to SAVING ON; “Off” to switch off the lights; and “Remote_On/Remote_Off” to gets into remote control mode ON/OFF;
  ○ *Status*: Returns the last measurements (Active/Reactive Power, Voltage and Current Intensity) available from the indicated device (Power controller or AMMS).
  ○ *Node_Historic*: Retrieves the historic measurements (Active/Reactive Power, Voltage and Current Intensity) of the device identified, between the dates (and time) pointed or between the date (and time) pointed and present time.

#### 5.2.5. Prototype Applications

Finally, for this prototype, two applications have been developed making use of this services API: the Presence Detection Software and the Simple Management Console.

The Presence Detection Software gathers the data coming from the light sensors and the presence detectors. Then, it takes the current status of the power controller and feeds its internal pedestrian detection algorithm, which decides when to send a command to the power controller to activate the saving mode or set the maximum illumination level.

**Figure 5.**
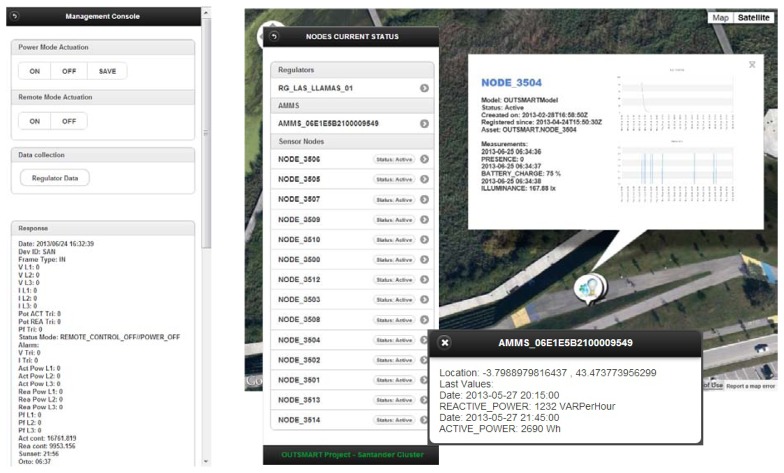
Power controller management console web interface to show current nodes status and historic graphic data (light levels and presence detections).

As shown in Figure 5, the Management Console shows information about the light levels detected and presence detection evolution and allows direct operation over the streetlights.

### 5.3. Real-World Prototype Deployment

From the point of view of Future Internet, the idea of this prototype is to show how a legacy system, belonging to utilities, can be integrated into IoT world. From the perspective of smart cities, this integration allows developers (and utilities themselves) to interact with these new nodes, combine these new capabilities with existing sensor networks and deploy new services, oriented to efficiency improvement, city sustainability and environment.

#### 5.3.1. Deployment Scenario

As a proof of concept of the aforementioned use case, a prototype has been developed and deployed.

**Figure 6.**
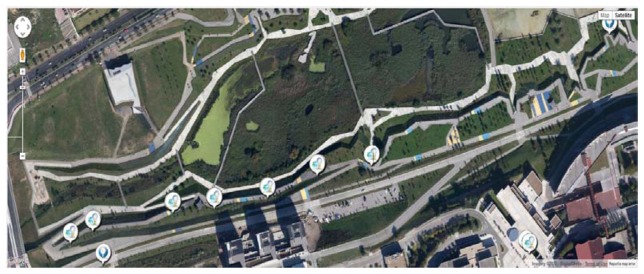
Las Llamas city park prototype deployment area.

Figure 6 shows the Las Llamas Park in Santander where the prototype has been deployed.

**Figure 7.**
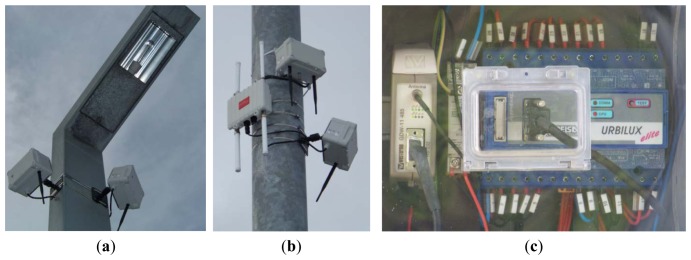
(**a**) Smartboxes attached to a streetlight; (**b**) Gateway deployed close to smartboxes; (**c**) Power controller's communication module and intelligent unit.

Figure 7 shows a detail of the different hardware devices installed for the prototype.

#### 5.3.2. Deployed IoT Devices

Prototype IoT Nodes, so-called smartboxes, integrate a number of devices that address the different installation requirements. These devices include several components that can be categorized by their main functionality as electrical components, communication components and sensors related components:

- The electrical components are related to the power storage and feeding of the device. These components are mandatory in any deployment for protecting the electric network from any smartbox malfunction. Electric components include a cable for charging the batteries, a residual current circuit breaker to protect the street light power system from a shortcut (or the device from external damage in case of electric storm), a battery which provides up to 2 days of autonomy and a power regulator.
- Communication components installed in order to provide wireless communication with the gateway; the prototype box includes a microprocessor, an XBee transceiver and an antenna. These communication components allow the smartbox to participate in a mesh network with the rest of IoT Nodes in the prototype. The proprietary Digimesh protocol is used for this purpose.
- Regarding the sensors, each prototype smartbox integrates 2 different sensors using a PCB adaptation board [27], a light sensor from Intersil and a presence detector sensor from Parallax. More specifically, the light sensor is a ISL76671 [28] and the presence detector used an X-band radar presence detector [29].

**Figure 8.**
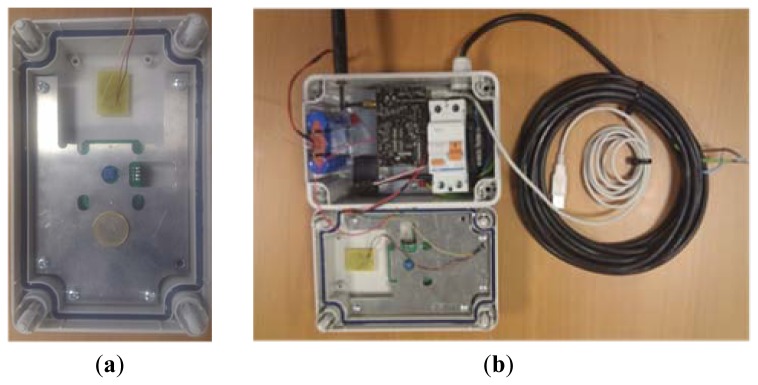
(**a**) Prototype smartbox cover where sensors have been mounted; (**b**) Prototype smartbox general view.

Figure 8 shows a detail of one of the IoT Nodes deployed for the prototype.

In the following sections we will describe the requirements and criteria that we used for the sensor selection. This includes quite useful information as pros and cons for several candidate sensors are provided extracted from real-world experience. Once analyzed the different options the option selected as the most appropriate is identified.

#### 5.3.3. Luminous Intensity Sensors

To understand the selection of the light sensor, it is important to note that we need to measure the *human eye response to illumination during late afternoon and night hours in outdoors environment*. This concept is referred as illuminance in photometry science and has the subjective connotation of being the human interpretation of light level.

Several options where considered for the prototype sensing needs:

- **LDR:** A photoresistor or light dependent resistor (LDR) is a semiconductor which electrical resistance decreases when exposed to direct light. This feature allows, when properly calibrated, measuring the light that is being received by the resistor.
- **Analog sensor based on a photodiode:** A photodiode is a photodetector that turns light into an electrical current or voltage; solar panels are based on this technology. The advantage of this technology over LDRs is that photodiodes have a wider linear light-current response, which means that it can be used for getting a more precise measurement as long as light radiation stays in the photodiode linear range. In this type of sensors, human eye response to light presence can be simulated by incorporating a specific filter to the sensor. This allows to measure illuminance.
- **Digital sensor based on a photodiode array:** This kind of sensors is based on the same technology of the above ones and incorporates the same filters for allowing detection of illuminance instead of Irradiance. The bigger difference is that such digital sensors incorporate a wide range of photodiodes, each of them with a linear response to illumination to different range of illuminance. In this way, by digitally selecting the diode, it is possible to measure light in a much wider range than using the analogue ones.

Table 1 shows the sensors that have been considered for their integration with the prototype.

**Figure 9.**
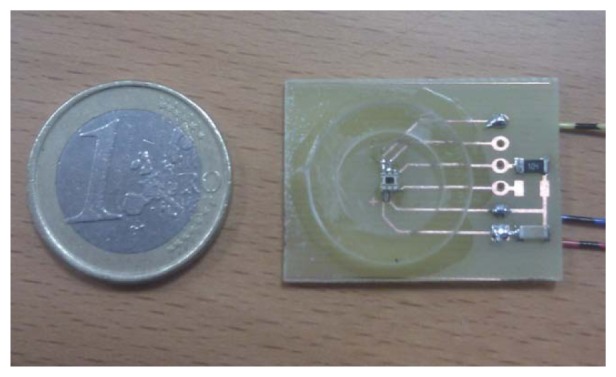
Prototype's built light sensor (ISL76671).

Figure 9 shows the ISL76671 sensor. This sensor was the one selected due to its behaviour under the night conditions in which the prototype would need to operate.

LDR sensors were not selected for two main reasons. First, they provide an objective assessment of the light radiation. Such physical magnitude takes the name of irradiance. Second, LDRs are more suited for detecting if current illumination is above or below any given threshold but not for detecting the amount of light present. This behaviour makes them a good solution when it is necessary to know if its day or night or if some indoor room has the lights turned on or off but not for measuring the amount of light with precision. Although digital options provided features beyond the once needed in the prototype deployment they were discarded since its cost were not justified.

#### 5.3.4. Presence Detection Sensors

Concerning the presence detection sensors, the design can be based on changes on echoes to emitted signals (active sensors) or changes in the detected neighbourhood (passive sensors).

This fact makes that presence detection is therefore based on movement detection. If any object of any size remains still for enough time, it will eventually conceal to the presence sensor until it starts moving again.

All presence sensors need to be calibrated, some of them are calibrated during manufacturing process and such calibration cannot be changed, while others accept some tuning.

Most off-the-shelf sensors have been designed for indoor conditions and therefore have been calibrated for working in such conditions. However, outdoor conditions are different because sensors have a wider line of sight and in most scenarios there will be something moving in the area, it might be the wind causing some tree or grass movement, could be animals, persons or even vehicles out of the sensor range.

Several options have been considered for the prototype sensing needs:

- **Computer vision presence detection:** it is possible to detect presence by installing a video camera and use specific computer vision analysis for detecting if there is human presence in the area. This kind of sensors has several advantages: they are able to detect presence in wider areas (as long as there is a free line of sight), they are able to detect several other things at same time and it is possible to tune the detection pattern (what is considered presence and what is not) depending on a wide range of parameters. This technology has some serious drawbacks; being a lot expensive, requiring a high bandwidth for communications, having problems to handle blind spots produced by trees or other big objects, having problems when operating under low illumination conditions and to require permanent power feeding for their operation.
- **Passive Infrared (PIR) sensor:** PIR sensors constantly measure the amount of heat (infrared radiation) received from the neighbourhood, when that radiation changes substantially they consider that there is movement in the area.
  This kind of sensors usually integrates a specific capsule that allows infrared radiation to reach the sensors, that capsule is usually a lent that focuses the ambient infrared radiation to the actual sensing device. Without such capsule the sensor would not be able to provide reliable measurements.
  Integrating them in any outdoors deployment requires a careful encapsulation for protecting them against weather conditions while allowing infrared radiation reach the sensor.
  This kind of sensors allows no tune on their decisions about if there is presence in the area or not since it is established during the manufacturing process.
  This technology detection range goes from 3 to 10 m as stated by the datasheets provided by various manufacturers.
- **Passive infrared (PIR) plus ultrasonic sensor:** an enhancement over PIR based sensors is to incorporate a ultrasonic sensor to them, this kind of technology combines a passive sensor (PIR) with an active sensor (ultrasonic) which allows to drastically reduce errors in detection while increasing sensor range.
  This kind of sensors has the same integration problems than the PIR ones: they require a specific encapsulating that allows infrared radiation to reach the PIR sensor while allowing ultrasonic sound to go through. The capsule must be able to protect the sensor from outdoors weather conditions. Commercial solutions for this technology are almost always designed for indoors conditions where it is possible to deploy them with relative cheap encapsulation. This kind of sensors allows no tune on their decisions about if there is presence in the area or not since it is established during the manufacturing process.
  This technology detection range goes from 3 to 12 m as stated by the datasheets provided by various manufacturers.
- **X-band RADAR presence detector:** X-band radars are able to detect human presence because of the wave length they operate (which is the one used for taking radiographies of bones). They operate by sending waves of X-band pulses and checking the Doppler effect of the echoes.
  In principle this would allow not only to detect presence but to detect the speed of the target too. That feature is not implemented in our prototype because (as specified by the manufacturer) that speed detection is only possible if there is only one target moving in the area of detection and it is moving straight to the radar sensor.
  This kind of technology allows tuning the calibration as it is possible to change the amount of time that the radar is sending and listening to the echoes and the amount of movement (or speed) that is set as threshold for triggering the detection signal.
  This technology detection range goes from 3 to 12 m as stated by the datasheets provided by various manufacturers.

Taking all this into account, we considered the sensors in Table 2 to be incorporated in the prototype:

The three sensors presented in Table 2 were tested. The objective of the tests was to identify the viability or not of using each of the sensors in outdoors environment. In that sense, the number of false positives and false negatives under prototype environment was analysed. It was also compared with the sensor behaviour when operating under laboratory conditions. Additionally, the detection range of the sensors was also assessed on another set of tests.

An unexpected feature discovered during those tests was that the RADAR sensor not only provides information on the presence of a moving object in the area, but also a measurement of the amount of movement detected. Additionally, RADAR sensor Doppler Effect translate into a measurement of presence detected when there is more than one target in the area.

This turned into a great advantage for the RADAR sensor. Additionally, the capacity to change the duration of the pulses and listening time, allowed fine grained adjustment of what is considered detection of presence.

An additional advantage of the X-band RADAR signal is that it is able to go through the smartbox that contains it, this makes the integration of it into the deployment much easier than any other sensor that would require an specific encapsulation. In this way RADAR sensors also reduce the visual impact of the deployment.

**Figure 10.**
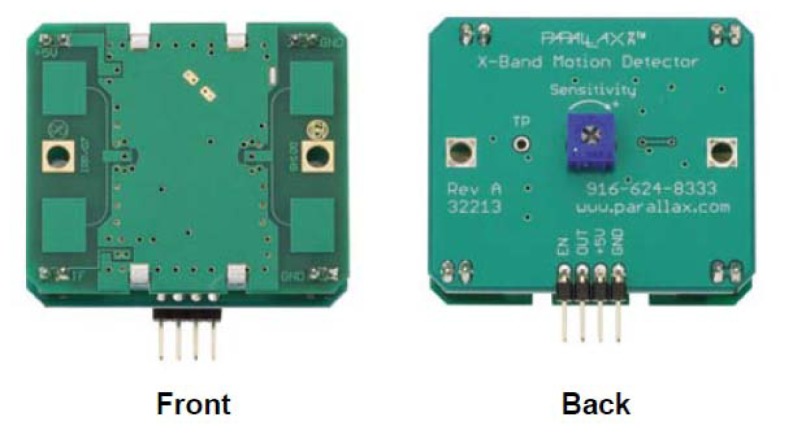
Prototype's built in RADAR.

Parallax X-band motion sensor, shown in Figure 10, was the selected device for the prototype deployment because of its better overall test results.

Computer vision presence detection was rejected because of its high communications requirements and its high energy consumption.

PIR sensors and PIR plus ultrasonic sensors were rejected due to their low overall performance when compared with RADAR detector.

#### 5.3.5. Power Controller and Lamps Restrictions

**Figure 11.**
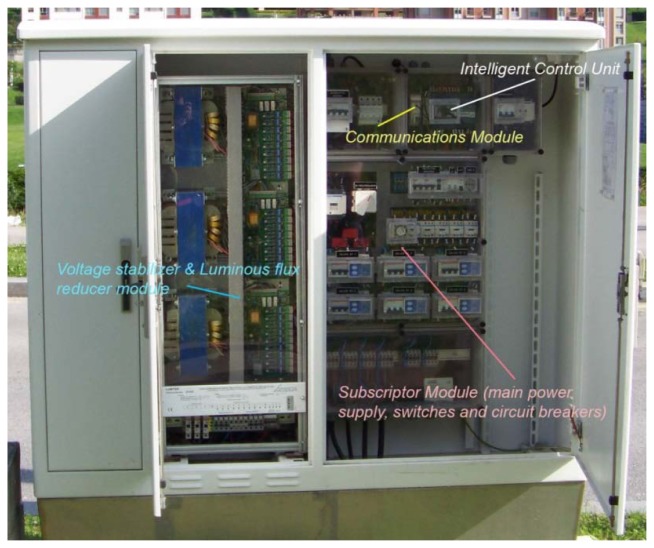
Prototype's power controller.

As it can be seen in Figure 6, the deployed prototype covered one part of the Las Llamas Park whose public lighting is supported by only one power controller (see Figure 11). From the point of view of the prototype, this power controller works as both, a sensor and an actuator.

As a sensor, it has been programmed to send, every 10 minutes from dusk till dawn, measurements of the provided active power (W), reactive power (VAr), triphase Voltage (V) and electric current intensity (mA).

As an actuator, it can process commands to switch on/off the streetlights, to set the power supplied to normal/saving mode, to activate/deactivate its astronomical clock and to retrieve a complete set of management data.

Continuing with this vision, the device can be divided into three main modules:

- *Intelligent Control Unit*, consisting of an *URBILUX ÉLITE RS 485 GSM 0-2V* [34] (made by *Arelsa*) device, implements the astronomical clock, the command processor to interact with controlling module and the reading & recording function. This element registers electrical parameters, measurements (which can be programmed to be taken up to every 15 s), events and alarms. This Urbilux also provides a RS-485 port to be connected to other control or information systems and a RS-232 port to connect with the communication module.
- *Communication module*, composed by a *Westermo GDW-11 485 GSM modem* [35], designed to allow connection of legacy RS-232, RS-422 and RS-485 serial equipment. Once connected to the intelligent module, provides the remote controlling functionality, allowing the Power Controller to receive commands from a remote operator and to send data as an IoT node. The prototype uses this GSM available modem but it could be easily replace by other communications gateway, supporting other protocols such UMTS, WiFi or ZigBee. This AT command driven modem operating over the GSM network provides speed up to 14.4 kbit/s.
- *Power supply and controlling element* is composed by the *subscriptor module* which plugs directly in the utility's power supply and integrates the corresponding switches and circuit breakers, and the *voltage stabilizer and luminous flux reducer module* that provides the corresponding power output to the streetlights line. This whole element is directly operated by the intelligent control unit. The voltage stabilizer and reducer module corresponds to a *Lumiter LMB61 400V III* + *N* [36], made by *Ingequr* with a rated in voltage of 400 V, provides an output power of 45 KVA with a rated output voltage of 230 V +/−2, 5% +/−2V or 187 V −0%, +4% in saving mode for sodium lamps.

**Figure 12.**
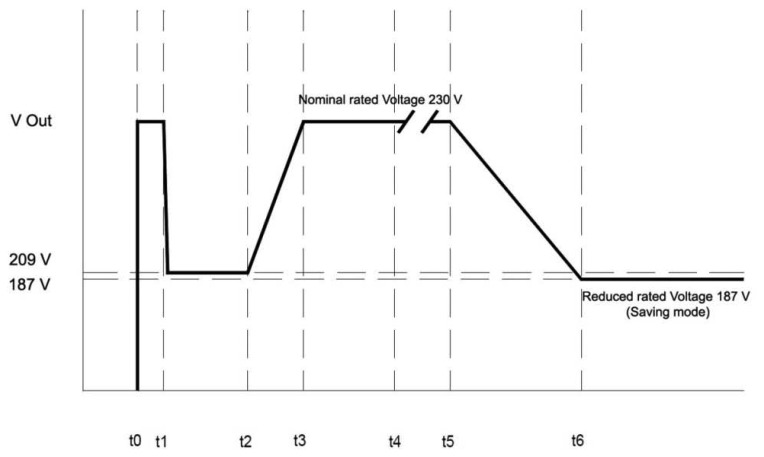
Time constraints to operate with voltage supplied.

The power controller operating mode has been configured to work with the currently installed high-pressure sodium lamps, which means several constraints in the way power must be supplied: any change to be made in the operating mode of the controller that concerns a change in the voltage supplied to the streetlights (including switching on/off the electrical supply) must obey the time slots shown in Figure 12 so the intelligent control unit implements an intelligent voltage guard circuit that prevents any actuation against this rule.

In practice, due to sodium lamps thermal stabilization (cf. Figure 12), we've established a 15 min guard time slot from the moment the power is switched on till the first command to change the operating mode (nominal to saving mode) can be sent and also, we will wait at least for another 15 min between changes (nominal to saving mode and *vs.*). Whether sodium lamps are replaced by led bulbs, these guard time slots could be omitted, and light adaptability would be done close to real time.

### 5.4. Autonomous Street Lighting Adaptability Application

The application layer communicates with the resources provided by the platform through the service layer in order to create functionalities that answer to real world demands. In this way, while the service layer provides access to the platform resources, the application layer is the one that deploys the actual functionalities that users will perceive.

Autonomous Public Street Lighting Adaptability application focuses on modifying the light provided by controlling the streetlights power. That power control will be based on the detection or not of presence in the area. In that way the application will keep the streetlights at their minimum power level, increasing it when there is activity in the area. For doing that the application must subscribe to the presence detection service. That service will send information to the application each time that some change happens in the presence detection pattern.

The Autonomous Public Street Lighting Adaptability application must then evaluate the detection pattern and decide if such pattern requires of any action on its side. For doing that the application must rely on an algorithm that must contemplate any possible technical restriction of the infrastructure.

Proof of concept algorithm checks for *presence detection on any pair of consecutive sensors in a time span no longer than 5 s*. In that way when any of the presence detector patterns change to “presence detected”, the algorithm checks if any other adjacent sensor is detecting presence or if it did in the past 5 seconds; then checks the current power provided to the streetlights. If based on that information an action is requested then the application checks if it is possible to take such action; if it is, then it increases the power provided to nominal value.

The application also receives notifications on sensors that are no longer detecting presence. When conditions for keeping power at nominal levels are not meet, the algorithm will reduce power to saving levels.

Due to the streetlights' technical restrictions described before, it is possible that no action can be taken, in this case the application will discard last data received and wait until next data arrives starting the algorithm again from zero.

When the algorithm determines that an action must be taken it will access the actuation service provided by the service layer of the platform. That service will then forward the petition to the IoT Backend Device Management enabler and from there the command is sent to the power regulator.

It's important to note that from the application level perspective everything happening below the service layer is completely transparent. This allows the easy integration of new applications because they only need to interact with the available services.

## 6. Conclusions

This paper provides insights on the way FI technologies create the necessary glue and logic that allows the integration of current vertical and isolated city services into a holistic solution. It has presented the functional architecture that harmonizes the different requirements imposed by the smart city scenario. In this sense we have identified the key design principles that have been addressed providing motivation and rationale for the proposed architectural design.

Moreover, the paper describes a real-world prototype, that instantiates the aforementioned architecture. This prototype has been deployed in a real-world environment and targets an application of FI technology with large savings opportunities.

An in-depth description of the smart city platform implementation details has been provided. Additionally, the actual deployment details have been also thoroughly presented allowing the assessment and discussion of potential adoption paths for the proposed solution into larger scale trials.

The initial experiences carried out with the implemented prototype are showing promising results and allowing the derivation of lessons learnt and important feedback that helps enhancing the reliability of the prototype and the energy savings achieved through it.

## Figures and Tables

**Table 1.** List of evaluated light sensors.

**Sensor**	**Distributor**	**Type**	**Reference**
Waspmote light sensor	Libelium	LDR	[27]
ISL76671	Intersil	Photodiode, Analog	[28]
ISL29023	Intersil	Photodiode, Digital	[30]
TSL2561	Taos	Photodiode, Digital	[31]

**Table 2.** List of presence sensors tested.

**Sensor**	**Distributor**	**Type**	**Reference**
PIR Sensor (#555-28027)	Parallax	PIR	[32]
Dual Motion Detector (reference: 75 15 60)	Conrad	PIR + ultrasonic	[33]
X-Band Motion Detector (#32213)	Parallax	Xband presence detector	[29]

## References

[1] City B.L., Assessment E. (2010). Urbanization and health. Bull. World Health Organ..

[2] Schaffers H., Komninos N., Pallot M., Trousse B., Nilsson M., Oliveira A. (2011). Smart cities and the future internet: towards cooperation frameworks for open innovation. The Future Internet.

[3] Hernández-Muñoz J.M., Vercher J.B., Muñoz L., Galache J.A., Presser M., Gómez L.A.H., Pettersson J. (2011). Smart cities at the forefront of the future internet. The future internet.

[4] Giffinger R., Fertner C., Kramar H., Kalasek R., Pichler-Milanovic N., Meijers E. (2007). Smart cities—Ranking of European medium-sized cities. http://www.smart-cities.eu/download/smart_cities_final_report.pdf.

[5] Strickland E. (2011). Cisco bets on South Korean smart city. IEEE Spectrum.

[6] IBM Intelligent Operations Center. http://www-142.ibm.com/software/products/us/en/intelligent-operations-center.

[7] Telvent Smart Cities Solutions. http://www.telvent.com/smartcities/smart_integration/index.cfm.

[8] Kloeckl K., Senn O., di Lorenzo G., Ratti C. Live Singapore!—An Urban Platform for Real-Time Data to Program the City.

[9] Amin S.M., Wollenberg B.F. (2005). Toward a smart grid: power delivery for the 21st century. IEEE Power Energy Mag..

[10] Chen S.Y., Song S.F., Li L., Shen J. (2009). Survey on smart grid technology. Power Sys. Technol..

[11] Gellings C.W. (2009). The smart Grid: Enabling Energy Efficiency and Demand Response.

[12] Ipakchi A., Albuyeh F. (2009). Grid of the future. IEEE Power Energy Mag..

[13] FI-WARE, Future Internet Core Platfom. http://www.fi-ware.eu.

[14] Competitiveness and Innovation Framework Programme (CIP) Smart Cities Project Portfolio. http://www.cordis.europa.eu/fp7/ict/fire/connected-smart-cities/csc_en.html.

[15] Sanchez L., Galache J.A., Gutierrez V., Hernandez J.M., Bernat J., Gluhak A., Garcia T. SmartSantander: The Meeting Point between Future Internet Research and Experimentation and the Smart Cities.

[16] Ballesteros J., Rahman M., Carbunar B., Rishe N. Safe Cities. A Participatory Sensing Approach.

[17] Roitman H., Mamou J., Mehta S., Satt A., Subramaniam L.V. Harnessing the Crowds for Smart City Sensing.

[18] Dutta P., Aoki P.M., Kumar N., Mainwaring A., Myers C., Willett W., Woodruff A. Common Sense: Participatory Urban Sensing Using A Network of Handheld Air Quality Monitors.

[19] Presser M., Gluhak A. The Internet of Things: Connecting the Real World with the Digital World. http://www.archive.eurescom.eu/message/messageSep2009/The-Internet-of-Thing%20-Connecting-the-real-world-with-the-digital-world.asp.

[20] Dunkels A., Vasseur J.P. IP for Smart Objects, Internet Protocol for Smart Objects (IPSO) Alliance. http://www.ipso-alliance.org/wp-content/media/why_ip.pdf.

[21] Toma I., Simperl E., Hench G. A Joint Roadmap for Semantic Technologies and the Internet of Things.

[22] Atzori L., Iera A., Morabito G. (2010). The internet of things: A survey. Comput. Netw..

[23] Sheng X., Xiao X., Tang J., Xue G. (2012). Sensing as a service: A cloud computing system for mobile phone sensing. IEEE Sens..

[24] Serbanati A., Medaglia C.M., Ceipidor U.B., Turcu C. (2011). Building Blocks of the Internet of Things: State of the Art and beyond. Deploying RFID-Challenges, Solutions, and Open Issues.

[25] Modbus I.D.A. Modbus Application Protocol Specification. http://www.modbus.org/specs.php.

[26] Telefonica IDAS' FI-WARE Wiki. http://www.forge.fi-ware.eu/plugins/mediawiki/wiki/fiware/index.php/IDAS.

[27] Waspmote Events Board Technical Guide http://www.libelium.com/development/waspmote/documentation/events-board-technical-guide/?action=download.

[28] Light Sensor ISL 76671 Data Sheet. http://www.intersil.com/content/dam/Intersil/documents/fn77/fn7716.pdf.

[29] Presence Sensor Parallax 32213 Specifications. http://www.parallax.com/Portals/0/Downloads/docs/prod/sens/32213-X-BandMotionDetector-v1.1.pdf.

[30] Light Sensor ISL29023 Data Sheet. http://www.intersil.com/en/products/optoelectronics/ambient-light-sensors/light-to-digital-sensors/ISL29023.html.

[31] Light Sensor TSL2561 Specification. http://www.adafruit.com/datasheets/TSL2561.pdf.

[32] Presence Sensor Parallax 555-28027 Specifications. http://www.parallax.com/Portals/0/Downloads/docs/prod/audiovis/555-28027-PIRsensor-v1.4.pdf.

[33] Presence Sensor Conrad 751560 Specifications. http://www.produktinfo.conrad.com/datenblaetter/750000-774999/751560-an-01-ml-Dualer_Bewegungsmelder_de_en_fr_nl.pdf.

[34] Arelsa's Urbilux Elite Specifications. http://www.construmatica.com/archivos/27716/control_cuadro_urbilux_elite.pdf.

[35] Westermo GDW-11 485 GSM Modem Specifications. http://www.westermo.com/web/web_en_idc_com.nsf/alldocuments/D040F51B97FB0D60C12578930034598C.

[36] Ingequr's Lumiter LMB61 400V III+N Voltage Stabilizer Specifications. http://www.ingequr.com/descargas/Doc_Tec_Equipos.pdf.

